# Can the Remaining Coronal Tooth Structure Influence the Mechanical Behavior of Nonpost Full Crowns?

**DOI:** 10.1055/s-0043-1776117

**Published:** 2024-03-31

**Authors:** Alana Barbosa Alves Pinto, Guilherme Schmitt de Andrade, Amjad Abu Hasna, Joyce Rodrigues de Souza, João Paulo Mendes Tribst, Alexandre Luiz Souto Borges

**Affiliations:** 1 Department of Dental Materials and Prosthodontics, Institute of Science and Technology of São José dos Campos, São Paulo State University (UNESP), São José dos Campos, São Paulo, Brazil; 2 Center of Biological and Health Sciences, School of Dentistry, Western Paraná State University (Unioeste), Cascavel, PR, Brazil; 3 Department of Restorative Dentistry, Endodontics Division, Institute of Science and Technology, São Paulo State University (ICT-UNESP), Av. Eng. Francisco José Longo Avenue 777, São José dos Campos, SP, CEP 12245-000, Brazil; 4 Department of Reconstructive Oral Care, Academic Centre for Dentistry Amsterdam (ACTA), University of Amsterdam and Vrije Universiteit Amsterdam, 1081 LA, Amsterdam, the Netherlands

**Keywords:** crowns, endodontically treated incisors, fatigue, fracture resistance, finite element analysis.

## Abstract

**Objectives** This study investigated the impact of the remaining coronal tooth structure on the mechanical behavior of nonpost (NP) full crowns on endodontically treated maxillary central incisors.

**Materials and Methods** Forty bovine incisors with NP and 2-mm of ferrule were divided into four groups based on the remaining structure: complete 2-mm ferrule (NP-2), absence of mesial and distal ferrule effect (NP-BL), absence of buccal and lingual ferrule effect (NP-MD), and no ferrule (NP-0). The specimens underwent a stepwise stress fatigue test until fracture occurred, and stress distribution was analyzed using in silico finite element analysis (FEA). Additionally, groups with endodontic posts (P) were simulated in the FEA.

**Results** The results showed that the survival rates varied among the different groups under oblique loading. The NP-2 group exhibited the highest survival rate, with all samples enduring loads up to 200 N and some surviving up to 520 N. The NP-MD and NP-BL groups had lower survival rates, while the NP-0 group had the poorest survival rate. The predominant failure mode was a nonrepairable root fracture. FEA results indicated no significant difference between groups with and without posts. NP intraradicular restorations on nonweakened roots with a minimum height of 2mm and partial or total ferrule thickness of 1mm offer a promising treatment option.

**Conclusion** A complete 2-mm ferrule was found to be the most favorable configuration for NP full crowns. However, maintaining the remaining tissue is crucial, as both combinations with preserved ferrule effect exhibited superior behavior in terms of fatigue and fracture load compared to the group with no ferrule. These findings contribute to understanding the mechanical considerations for NP full crowns and provide insights into treatment planning and design choices in restorative dentistry.

## Introduction

The preparation of a post and core in dental procedures carries certain inherent risks, including root perforation and excessive widening of the root canal during preparation. 1 2 On the other hand, opting not to install a post allows for reversibility and preservation of the tooth structure. 3 It is important to note that post and core procedures do not possess the capability to reinforce the remaining tooth structures. 3 4 5 This limitation stems from their higher elastic modulus (40 GPa) compared to that of the root dentine tissue (18 GPa), resulting in an unnatural biomechanical behavior of the restoration. 6

The prognosis of prosthetic treatment for endodontically treated teeth (ETT) is directly influenced by the amount of remaining tooth structure, known as the ferrule effect. 7 A 2-mm margin of healthy tooth structure is recommended to provide the necessary ferrule effect, which helps protect the root against vertical fracture. 7 8 Additionally, the direction of occlusal loading can also impact the longevity of restorative treatments. 8 9 Although posterior teeth experience higher load intensity compared to anterior teeth, failures in extensively compromised anterior teeth often occur due to the concentration of tensile forces generated by vertical components rather than compression forces. 10

In this regard, studies have shown that the non-post restorative approach for posterior teeth appears promising even without a ferrule, likely due to the axial loads they experience. 2 11 However, obtaining a complete or partial ferrule with a minimum height of 2 mm is not always achievable in clinical practice due to extensive caries or coronal fractures. In such cases, orthodontic extrusion or clinical crown lengthening surgery is recommended to achieve the required height, but both methods have their drawbacks. Enlarging the clinical crown can impact aesthetics by altering the levels of the gingival margin, and orthodontic treatment entails significant time, cost, and patient discomfort. Additionally, both treatments result in a decrease in the length of the root anchored in the alveolar bone, thereby reducing periodontal support. 12 13 14

Although incomplete ferrule cases are more commonly encountered in clinical practice, there is controversy surrounding the impact of the location of the remaining tissue on the mechanical behavior of ETT, as indicated by previous studies. 12 15 Furthermore, several studies have examined the effect of ferrule design in the presence of posts, which has been shown to have a negative influence on the failure mode. 3 8 16 Consequently, there is an increasing need for further research aimed at understanding the impact of the ferrule and the location of the remaining walls in teeth without intraradicular retainers, particularly when exposed to horizontal loads.

This study aimed to evaluate the effect of the residual ferrule of the remaining coronary structure on the biomechanical behavior and fatigue test of crowns on endodontically treated central incisors. The null hypothesis is that (1) there is no difference in mechanical behavior between the groups with partial and/or total ferrule regardless of the presence of post and (2) there is no difference in fatigue survival between the groups with partial and/or total ferrule regardless of the presence of post.

## Material and Methods

### Finite Element Analysis

**Fig. 1:**
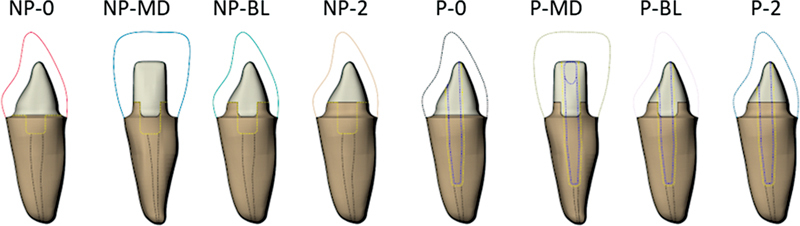
Evaluated models according to postendodontic restorative approach: no post and no ferrule (NP-0); no post and 2-mm ferrule at proximal walls (NP-MD); no post and 2-mm ferrule at buccolingual walls (NP-BL); no post and complete 2-mm ferrule (NP-2); post and no ferrule (P-0); post and 2-mm ferrule at the proximal walls (P-MD); post and 2-mm ferrule at the buccolingual walls (P-BL); post and complete 2-mm ferrule (P-2).

A previously validated three-dimensional (3D) model of an upper central incisor was designed in a 3D format containing enamel, dentin, and cement, and was simulated as ETT. 17 18 The factorial design considered the factors: presence and location of the remaining tissue in four levels with post (P) and no post (NP). The evaluated groups are summarized in Fig. 1 .

All models were geometrically exported in Standard for the Exchange of Product (STEP) model data to computer-aided engineering (CAE) software (ANSYS 20.0, ANSYS, Houston, TX, United States) for a static structural mechanical analysis. Static analysis is an effective way to predict the behavior of structures and systems in controlled experiments, used when load conditions are predominantly constant or when their variation occurs slowly. The static analysis calculates deformation, stress, and displacement in response to loads; furthermore, the results obtained from it can be directly compared with *in vitro* findings, allowing for direct validation of the results.

All contacts were considered bonded, and the models were fixed on the bottom surface of the cylinder. All models were loaded with 400 N at an angle of 30 degrees 2 mm above the cingulum (load application point). The materials were considered homogeneous, linearly elastic, and isotropic, except for the fiberglass posts, which were considered orthotropic ( Table 1 ). After the mesh convergence test at 10%, it obtained an average of 72,719 tetrahedral elements and 128,756 nodes. The stress distributions were evaluated by the maximum principal stress (MPS) criterion following the *in vitro* test parameters, described in the following section.

**Table 1:** Mechanical properties considered in this study

Material	E (GPa)	G (GPa)	Ѵ
Acrylic resin	2.7	–	0.35
Dentin	18.6	–	0.32
Indirect composite resin	14.8	–	0.30
Resin cement	6	–	0.28
Direct composite resin	21.62	–	0.24
Fiberglass post	X = 37 Y = 9.5 Z = 9.5	Xy = 3.1 Xz = 3.5 Yz = 3.1	Xy = 0.27 Xz = 0.34 Yz = 0.27

Abbreviations: GPa, Gigapascal.

## Fatigue Behavior

### Experimental Design

The study categorized the experimental groups into four distinct levels, each representing a different configuration:

- Complete 2-mm ferrule (NP-2): This group featured a complete 2-mm ferrule.
- Two-millimeter ferrule with buccal and lingual free walls (NP-BL): In this group, there was a 2-mm ferrule on the buccal and lingual walls, with the remaining walls being free.
- Two-millimeter ferrule in the proximal walls (NP-MD): This group had a 2-mm ferrule specifically on the proximal walls.
- No ferrule (NP-0): The last group, NP-0, lacked any form of ferrule.

### Teeth Selection

A total of 40 bovine incisors were included in the study based on their similarity in size and morphology. The buccolingual and mesiodistal widths of the teeth were measured using a digital caliper (Starrett 727, Starrett, Itu, Brazil), allowing a deviation of up to 10% from the mean. The teeth were thoroughly cleaned and examined under a stereomicroscope (Discovery V20, Carl Zeiss Microscope, Göttingen, NI, Germany) to identify any factors that could potentially affect the research outcomes, such as fractures, defects, or cracks. To preserve the bovine teeth until their use, a 0.1% thymol solution with a pH of 7.0 was employed and stored at 4°C.

The root portion of the teeth was standardized by sectioning it using a double-sided diamond disk (KG#7020, KG Sorensen, Cotia, Brazil) attached to a high-speed handpiece. The sectioning process was performed under continuous irrigation to maintain the integrity of the root. The root length was standardized at 16 ± 0.5 mm for the NP-2, NP-MD, and NP-BL groups, ensuring consistency among these groups. For the NP-0 group, the root length was standardized at 14 ± 0.5 mm, reflecting the specific requirements of that group.

### Biomechanical Root Canal Preparation and Obturation

The root canals were explored with no. 15 K-file (Dentsply Maillefer, Ballaigues, Switzerland) and irrigated with 2.5% sodium hypochlorite solution (NaOCl; Asfer, São Caetano do Sul, Brazil). Immediately thereafter, instrumentation was performed with an R50.05 file of the RECIPROC system (VDW, Munich, Germany) and irrigated with 5 mL of 2.5% NaOCl for each third. The working length was determined visually by backing out 1 mm of file length after reaching the apical foramen.

After the instrumentation, the canals were filled with 17% ethylenediaminetetraacetic acid (EDTA; Maquira, Maringá, Brazil) for 3 minutes and activated with the no. 30 K-file. Finally, the canals were washed out with 10 mL of saline solution dried with a no. 50 paper points, and filled with 50.05 Gutta-percha cones (VDW) and AH-Plus sealer (Dentsply Maillefer) using the lateral condensation technique.

### Embedding the Specimen

Each specimen was carefully placed and secured inside a polyvinyl chloride (PVC) tube with the assistance of a dental surveyor. The root assembly was fixed in place using Godiva Exata (Nova DFL, Taquara, Brazil). This ensured that the specimen was centered within the tube and that its long axis was perpendicular to the ground. The tube was then filled with self-cured acrylic resin (VIPI Flash, VIPI, Pirassununga, Brazil), and the entire setup was carefully centered within the tube, leaving approximately 4 mm of the cervical margin of the tooth exposed. To maintain the specimens in optimal condition, they were submerged in a closed container filled with deionized water at a temperature of 37°C until the next stage of the experiment.

### Preparing Remaining Walls

The buccal and lingual walls of the NP-MD group and the proximal walls of the NP-BL group were standardized using a conical diamond bur with rounded angles (#846KR Ø31 drill, Jota, Ruthi, Switzerland) with a high-speed handpiece and attached to a device for standardizing the preparations.

### Core Buildups and Full Crown Preparations

The cervical third of the root canal was widened with a diamond tip 846KR.025FG with a diameter of 3.1 mm (Jota) leading 3 mm from the root canal mouth.

The morphology of the filling cores was standardized using a master preparation for a full ceramic crown performed on an upper right central incisor of a typodont (P-Oclusa, São Paulo, Brazil) with a 1-mm-deep chamfer finish, 1-mm wear on the buccal, palatal, and proximal surfaces, and 2-mm incisal reduction. The master preparation was scanned by a CS 3600 scanner (Carestream Dental, Atlanta, GA, United States) and was printed through a 3D printer (Wilcos W3D, Petrópolis, RJ, Brazil) in UV resin (RESILAB3D, Petrópolis, RJ, Brazil) to obtain standardized dies. The dies were placed in an ultraviolet (UV) chamber for 150 seconds for complete curing of the material. Using these dies, acetate matrices were made and used to make the core fillings. For this purpose, a 1-mm-thick acetate plate (BioArt Equip Odontológicos, Ltda, São Carlos, Brazil) was used in a vacuum pressing machine (BioArt Equip Odontológicos, Ltda).

For the composite filling, the dentin of the coronal portion was etched with 37% phosphoric acid (Fusion Duralink, Angelus, Londrina, Brazil) for 15 seconds, washed for 30 seconds, and dried with absorbent paper. Two layers of Futurabond U adhesive (VOCO GmbH, Cuxhaven, Germany) were applied to the entire coronary portion, and the solvent was evaporated with an air jet and light cured for 10 seconds (Bluephase N, Ivoclar Vivadent, Schaan, Liechtenstein). The core buildups were made of VisCalor bulk fill resin (VOCO GmbH), which was heated to a maximum increment of 4 mm and light-cured for 10 seconds with a high-intensity LED (1,200 mW/cm ^2^ ; wavelength between 440 and 480 nm; Bluephase N, Ivoclar Vivadent) using the previously made acetate matrix.

In all groups, a preparation with a rounded shoulder (#446KR.12 Ø, Jota) with a depth of 1 mm was carried out, for the NP-2, NP-MD, and NP-BL groups. The purpose of the grinding was to obtain a 2-mm ferrule. In the NP-0 group, the termination was performed at the interface between the remnant and composite resin filling core.

## Crown Manufacturing

The preparation of each specimen was scanned using a CS 3600 scanner (Carestream Dental). To standardize the morphology of the crowns, an upper central incisor was scanned using an intraoral scanner (CS 3600, Carestream Dental) before the master preparation and then exported to the CAD software Inlab 3.80 (Sirona Dental Systems, Bensheim, Germany), which was used as a reference for the virtual design of each crown using the biogeneric copy technique. The Cerec InLab MC XL equipment (Sirona Dental Systems) was used to break the CAD/CAM composite resin blocks (Grandio blocs, VOCO GmbH).

## Luting Procedure

**Fig. 2:**
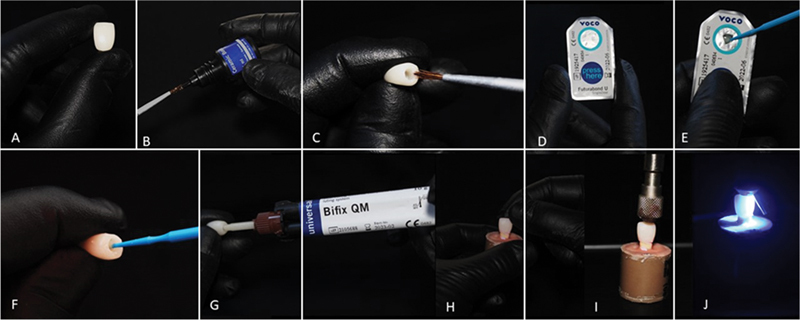
Luting procedure. ( **A** ) Grandio blocs crown, ( **B** ) ceramic primer being collected, ( **C** ) ceramic primer being applied inside the crown, ( **D** ) adhesive Futurabond U, ( **E** ) squeezing the blister and collecting the adhesive with microbrush, ( **F** ) active application of adhesive inside the resin crown, ( **G** ) injecting Bifix Gm cement inside the crown, ( **H** ) placement of the crown into position with the luting material, ( **I** ) load application of 750 g, and ( **J** ) light curing on each side of the crown.

The crowns were cleaned in an ultrasonic bath with isopropyl alcohol for 3 minutes. Silane (Ceramic Bond, VOCO GmBH) was applied to the intaglio surface of the crown and left for 60 seconds. A thin layer of the dual-curing self-etching adhesive system Futurabond DC (VOCO GmBH) was actively applied for 20 seconds and dispersed with light air jets, then light-cured for 20 seconds (Bluephase N, Ivoclar Vivadent). Dual resin cement (Bifix QM, VOCO GmBH), using the self-mixing tip, was applied to the restoration, which was placed into position with the aid of an adapted surveyor with a 750-g load. All cement excess was removed, and the load was held in position for 5 minutes. Then five light cures every 30 seconds (Bluephase N, Ivoclar Vivadent) were performed on each face (buccal, palatal, occlusal, mesial, and distal). The specimens were kept in distilled water at 37°C for 24 hours. The steps for the luting procedure are illustrated in Fig. 2 .

## Fatigue Test

To determine the fatigue test parameters, three specimens from each group were submitted to the maximum load to fracture. The specimens were positioned at a 30-degree angle to the base of the universal testing machine (Emic DL 1000, Emic, São José dos Pinhais, PR, BR). Then, a compressive load was applied 2 mm above the cingulum, using a load cell of 1,000 kg and a speed of 1.0 mm/min. A stainless steel load applicator with a rounded tip corresponding to a 6-mm-diameter ball was used. The applied load was increased until the specimen was fractured. The average of the maximum load values (N) was used for each experimental group to determine the load steps for the fatigue test.

Analysis and fatigue failure load were obtained by the stepwise stress accelerated life test. 19 20 21 22 To perform fatigue simulation, the specimens were attached to the mechanical testing machine (Biopdi, São Carlos, SP, Brazil), and load application was performed in the same way as in the monotonic test. The samples were loaded until fracture. During the test, the specimens were kept immersed in distilled water. The presence of cracks and/or fractures was checked, using illumination, every 10,000 cycles. The number of cycles, the load, and the failure mode were recorded. The failure modes were evaluated according to the restorable and irreparable classification. 23

### Statistical Analysis

All data were tabulated in a survival table, analysis of variance (ANOVA), and Tukey's analyses were performed, followed by pairwise multiple comparisons, all at a 5% significance level (Prism7, GraphPad, La Jolla, CA, United States).

## Results

### Finite Element Analysis

After checking the consistency of the results by evaluating the total deformation and equivalent Von Mises stress in CAE software (Engineering Simulation & 3D Design Software - ANSYS), a comparison between the models was made using the MPS criterion and qualitative analysis employing a colorimetric scale at the cement–crown and cement/core interfaces, core, ferrule, and root dentin.

### Cement–Crown Interface

**Fig. 3:**
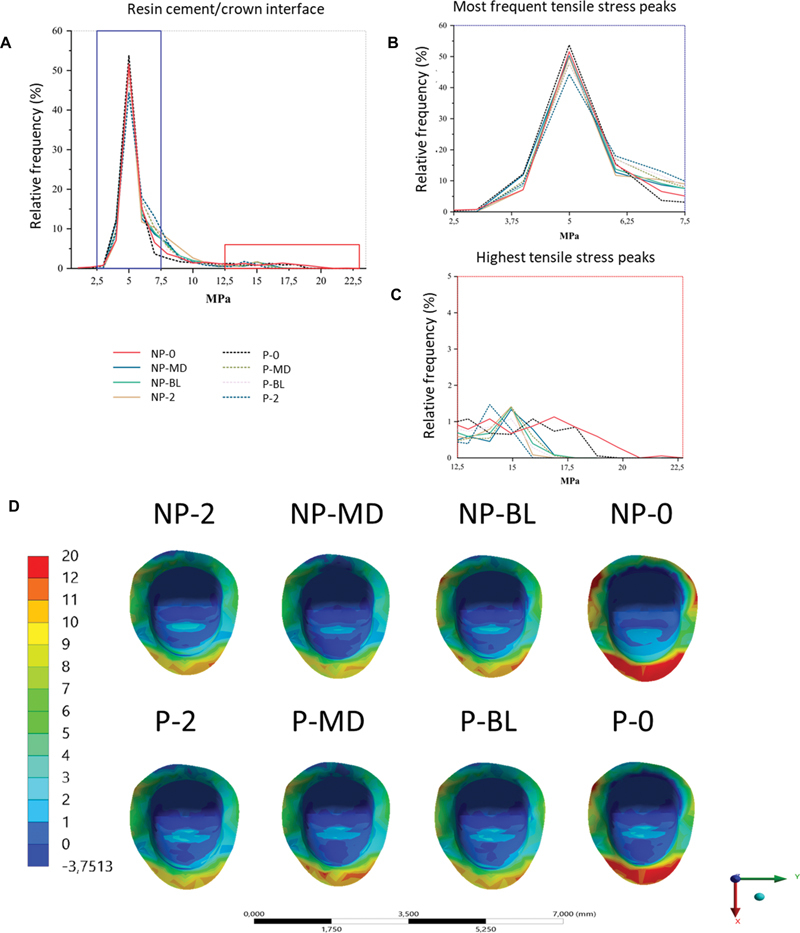
Stress distribution at resin/cement crown interface for all groups. ( **A** ) Scatter plot of data from the cement -crown interface. ( **B** ) Magnified area of most frequent stress peaks. ( **C** ) Magnified area of peak stresses of the greatest magnitude. ( **D** ) Stress distribution at the adhesive interface (resin/cement crown) according to each model. Non-post (NP), with post (P), no ferrule (0), two-millimeter ferrule in the proximal walls (MD), two-millimeter ferrule with buccal and lingual free walls(BL), complete 2-mm ferrule (2).

At the cement–crown interface region, around 50% of the adhesive interface of each group remained between 2.5 and 7.5 MPa. The stress peaks of greatest magnitude showed that the NP-0 group had the highest frequency of greatest magnitudes, with the highest peak around 22.5, followed by the P-0 group, with 19 MPa. In the P-MD, NP-MD, P-BL, NP-BL, and NP-2 groups, the magnitude of the frequency of the highest peaks was close. The P-2 group had the lowest peak value of MPS when compared to the other groups, with 16 MPa ( Fig. 3 ).

In the qualitative analysis of the groups, the NP-0 group showed the largest area under tensile stress among all groups, followed by the P-0 group. Next, the groups with partial ferrule in the mesiodistal region showed larger areas of stress, but the P-MD group showed larger areas of stress when compared to the NP-MD group. In the NP-2, P-2, NP-BL, and P-BL groups, the stress areas were smaller and similar to each other ( Fig. 3 ). The stresses were mainly concentrated in the region of the palatal margin of the preparation.

### Cement–Core Interface

**Fig. 4:**
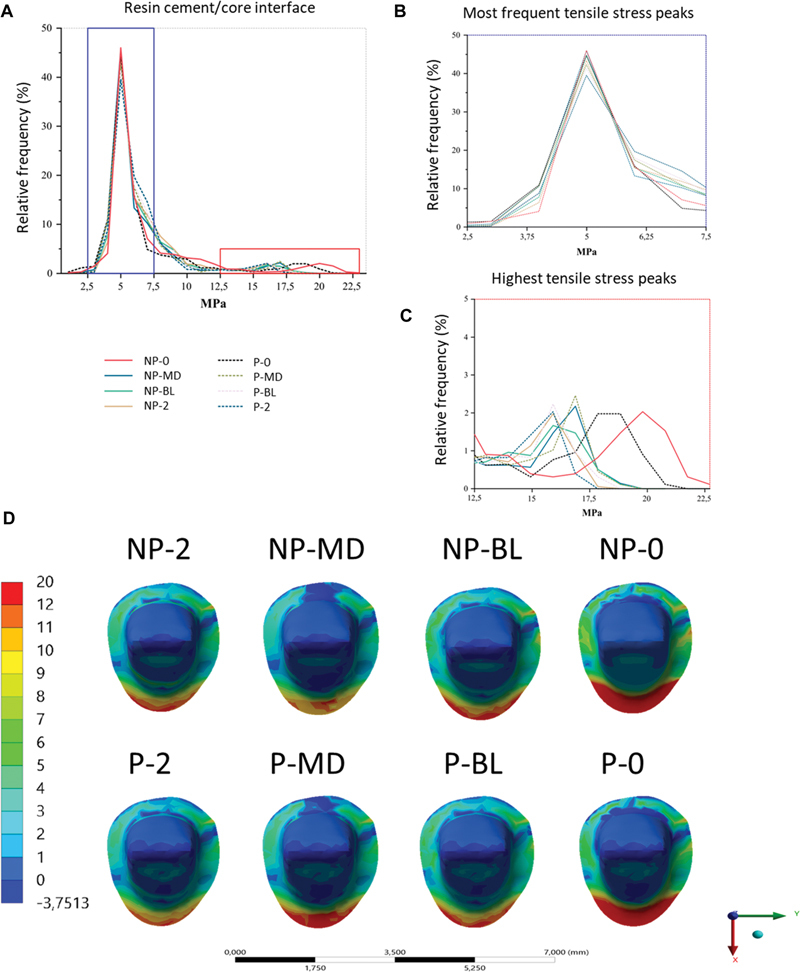
Stress distribution at cement/core buildup interface for all groups. ( **A** ) Scatter plot of data from the cement/core interface. ( **B** ) Magnified area of more frequent stress peaks. ( **C** ) Magnified area of peak stresses of greater magnitude. ( **D** ) Stress distribution at cement/core buildup interface for all groups. Non-post (NP), with post (P), no ferrule (0), two-millimeter ferrule in the proximal walls (MD), two-millimeter ferrule with buccal and lingual free walls(BL), complete 2-mm ferrule (2).

All groups showed tensile stress concentrations of 2.5 to 7.5 MPa, which represents an area of around 40 to 45.5% of the cement–crown interface. The NP-0 group showed higher peak stresses with greater magnitudes, with a maximum peak greater than 22.5 MPa, followed by the P-0 group with approximately 21 MPa, and with a higher frequency of 15 to 20 MPa. The P-MD and NP-MD groups had approximate peak frequencies, staying around 15 to 17.5 MPa. The P-BL, P-2, and NP-2 groups had approximate peak frequencies among themselves (2%) and lower peaks (15.5 MPa) when compared to the groups detailed above. The NP-BL group did not have a high frequency of larger peaks (<2%) but reached a higher peak tensile stress (19 MPa) than P-MD, NP-MD, P-BL, P-2, and NP-2 ( Fig. 4 ).

Qualitatively, the NP-0 and P-0 groups showed larger areas of stress concentration compared to the other groups. The P-BL group showed smaller stress areas when compared to the NP-BL group. The P-MD group showed areas of compression similar to the NP-MD group but with smaller maximum stress points in the palatal region. The NP-2 and P-2 groups showed approximate areas of stress concentration ( Fig. 4 ). The stresses were mainly concentrated in the region of the palatal margin of the preparation.

### Core Analysis

The largest area of tensile stress concentration occurred around 4 MPa for all groups but with different frequencies. This was most frequent for the NP-2 group (75.5%), followed by NP-0 (∼60%), NP-MD and P-2 (50%), P-0 and P-MD (45%), and P-BL (∼30%).

**Fig. 5:**
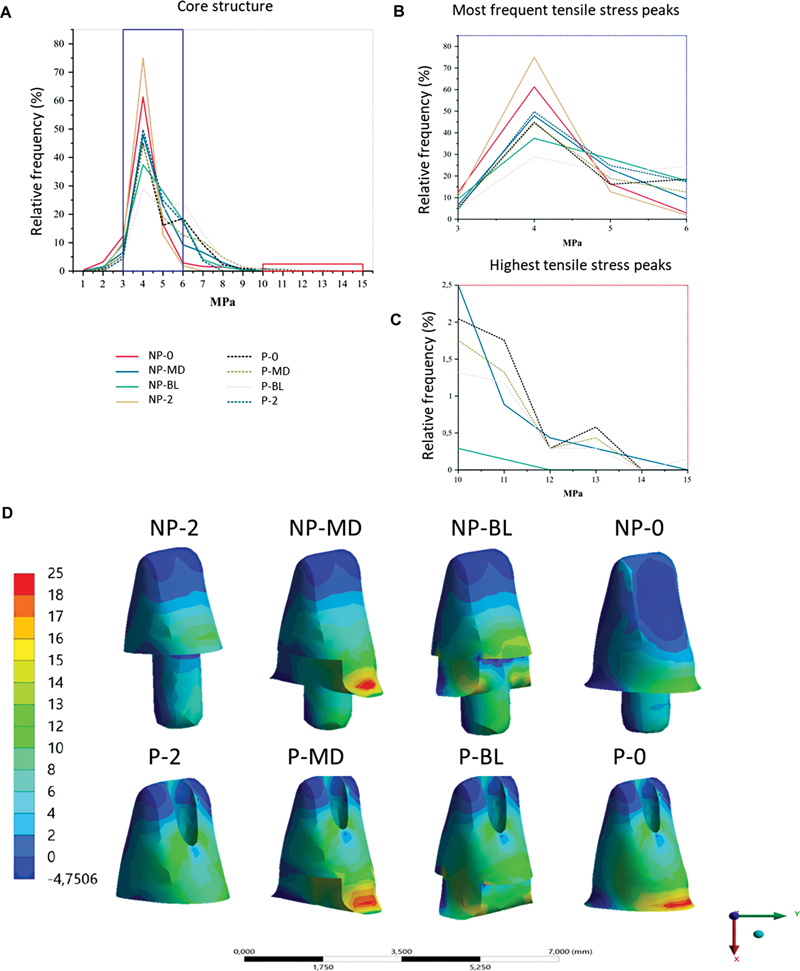
Stress distribution at core buildup interface for all groups. ( **A** ) Scatter plot of core data. ( **B** ) Magnified area of most frequent stress peaks. ( **C** ) Magnified area of higher magnitude stress peaks. ( **D** ) Stress distribution at core buildup interface for all groups. Non-post (NP), with post (P), no ferrule (0), two-millimeter ferrule in the proximal walls (MD), two-millimeter ferrule with buccal and lingual free walls(BL), complete 2-mm ferrule (2).

The maximum peak tensile stresses were 6 to 15 MPa, with the maximum peak being reached by P-BL and NP-MD, but P-BL held for higher magnitudes at higher frequencies (25% at 6 MPa, 1.8% at 10 MPa, and 0.5 at 15 MPa). The P-MD and P-0 groups maintained higher peak stress magnitudes but reached a maximum peak tensile stress of 14 MPa. The P-BL group reached a maximum of 12 MPa. The maximum peaks of the remaining groups were 8.5 MPa for NP-0, 8 MPa for P-2, and 7 MPa for NP-2 ( Fig. 5 ).

In the qualitative analysis, the geometry interferes with the stress distribution of the groups. The mesiodistal walls do not favor the direction of force application, causing the system to have lower compliance, which increases stress concentration, which is seen in the NP-MD, P-MD, and P-0 groups. The NP-BL and P-BL groups showed smaller areas of maximum stress when compared to the NP-MD, P-MD, and P-0 groups due to the stiffness of the components. The NP-0, P-2, and NP-2 groups had larger compression areas and fewer tension areas ( Fig. 5 ).

### Root Dentin

**Fig. 6:**
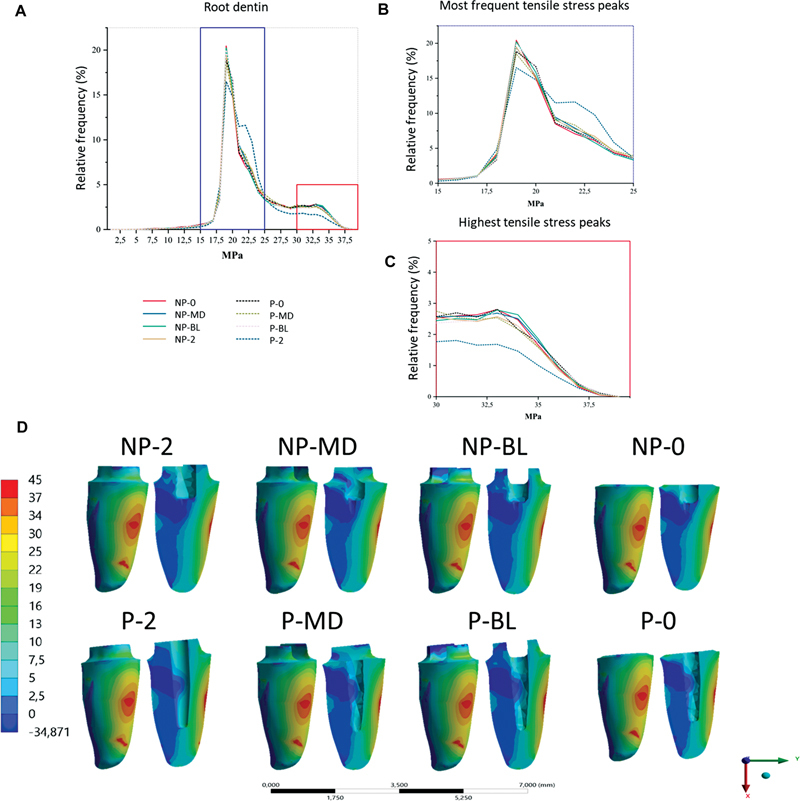
Stress distribution at root dentin for all groups. ( **A** ) Scatter plot of root data. ( **B** ) Magnified area of most frequent stress peaks. ( **C** ) Magnified area of higher magnitude stress peaks. ( **D** ) Stress distribution at root dentin for all groups. Non-post (NP), with post (P), no ferrule (0), two-millimeter ferrule in the proximal walls (MD), two-millimeter ferrule with buccal and lingual free walls(BL), complete 2-mm ferrule (2).

All groups showed a tensile stress concentration of 19 MPa with a frequency between 15 and 20% of all groups. In the analysis of MPS in the middle third of the root, all groups, except the NP-2 group, between 30 and 35 MPa had a frequency of 2.5%. The P-2 group had a frequency lower than 2% between 30 and 35 MPa. All groups reached peaks higher than 37.5 Mpa ( Fig. 6 ). In the qualitative analysis, all groups showed very similar areas of compressive and tensile stress in the middle-third root ( Fig. 6 ). However, when the root and core are analyzed together, the NP-BL group has larger regions of tension in the cervical region than the NP-MD group ( Fig. 6 ).

## Fatigue Test

The descriptive analysis of the results for loading and stepwise stress cycles is shown in Tables 2 and 3 .

**Table 2:** Results from fatigue analysis based on average fatigue failure load, median, and respective 95% confidence intervals, according to the different evaluated groups

Groups		Fracture load in fatigue				
				95% confidence interval		
	Average ( *N* )	Standard deviation	Tukey	Median ( *N* )	Lower bound	Upper bound
NP-0	252	99.9	B	200	190.1	313.8
NP-2	664	222.5	A	560	394.7	725.2
NP-BL	364	124.3	B	360	238.5	481.4
NP-MD	632	214.8	A	600	426.4	773.5

**Table 3:** Results from fatigue analysis based on the number of cycles for fracture in fatigue, median, and respective 95% confidence intervals, according to the different evaluated groups

Groups	No. of cycles for fracture in fatigue					
					95% confidence interval	
	Average ( *N* )	Standard deviation	Tukey	Median ( *N* )	Lower bound	Upper bound
NP-0	115.000	124.83	B	50.000	37.62	192.37
NP-2	630.000	278.08	A	500.000	293.39	706.60
NP-BL	255.000	155.36	B	250.000	981.790	401.82
NP-MD	590.000	268.53	A	550.000	333.06	766.93

**Fig. 7:**
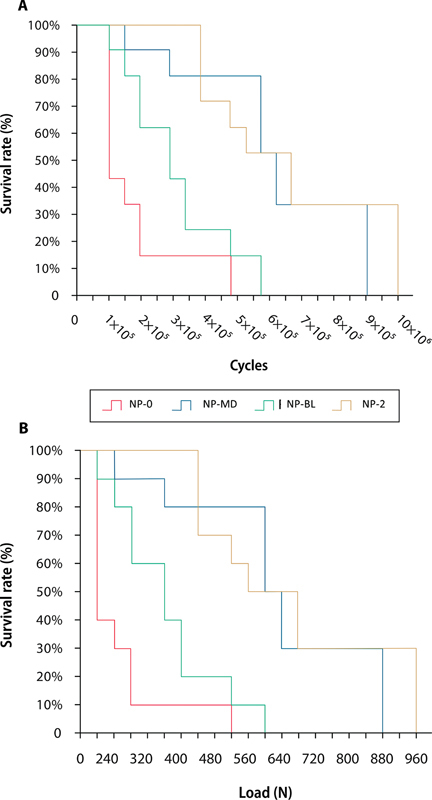
Survival graphs obtained by Kaplan–Meier and log-rank tests for ( **A** ) load to failure (MPa;) and ( **B** ) number of cycles for failure. Abbreviations: NP; Non-post, P; with post, 0; no ferrule, MD; two-millimeter ferrule in the proximal walls, BL; two-millimeter ferrule with buccal and lingual free walls, 2; complete 2-mm ferrule.

Tukey's statistics for the survival data as a function of oblique loading detected some statistical differences between the conditions analyzed ( Fig. 7 ). It could be evidenced that 100% of the samples survived loading up to 200 N. In all, 80% of the NP-MD and NP-BL groups survived 400 and 280 N, respectively. As for the NP-0 group, only 40% survived to 240-N loading. The NP-2 group, with the best survival rate, reached 440 N in 100% of the samples; subsequently, 50% survived 520 N.

In the graph of survival to fracture by cycles ( Fig. 7 ), there was also a statistical difference between the groups studied. The NP-0 group showed failure initiation of 60% of the samples around 50,000 cycles. Eighty percent of the NP-MD and NP-BL groups survived 250,000 and 150,000 cycles, respectively. The NP-2 group had a 100% survival rate of the samples up to 350,000 cycles, surviving 20% of the samples up to 950,000 cycles, and subsequently 30% up to a load of 960 N. However, 30% of the NP-MD group survived a load of 880 N, closely resembling the NP-2 group.

## Failure Mode

**Fig. 8:**
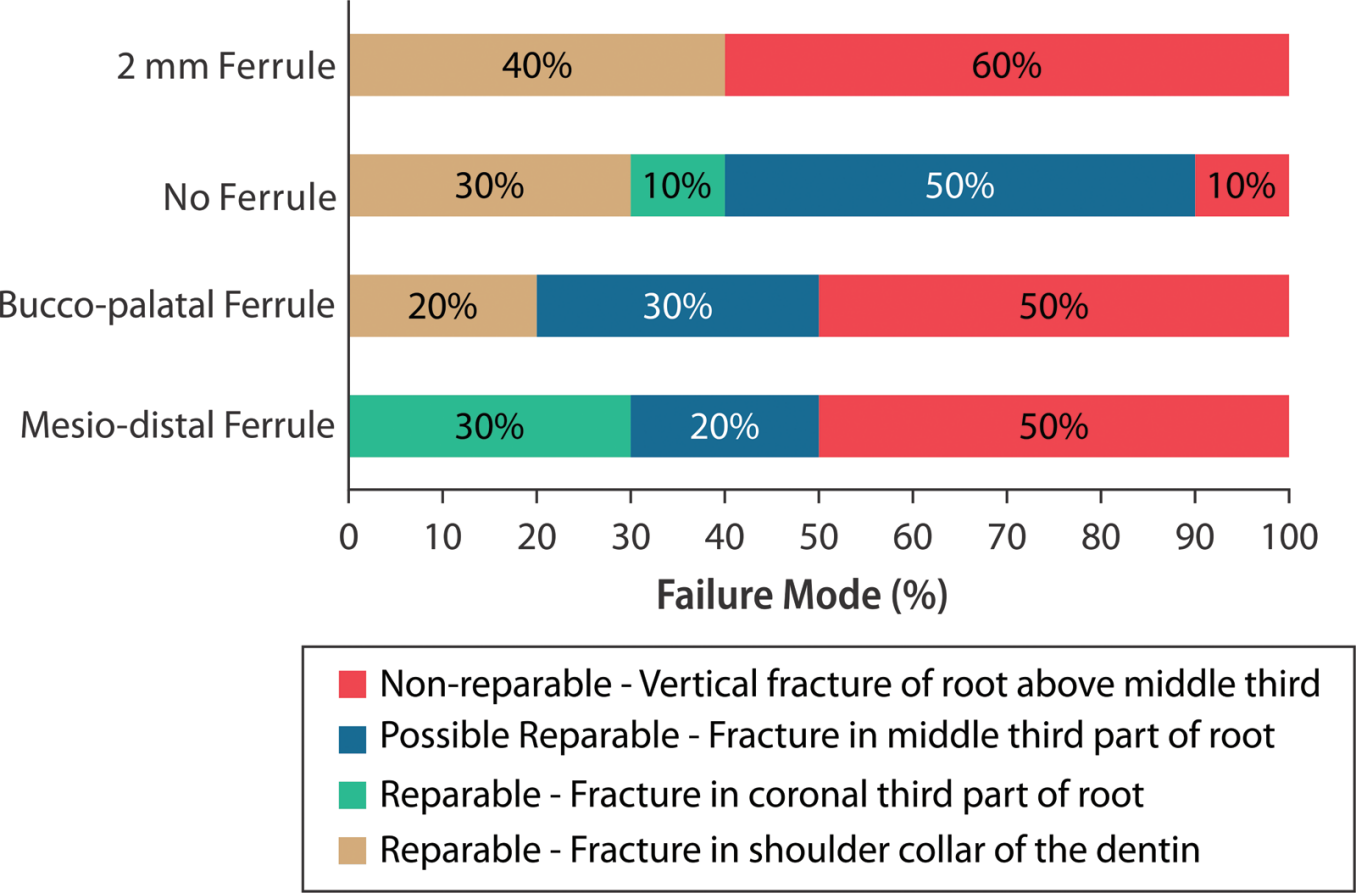
The classification of failure modes according to the percentage of incidence per group.

When evaluating failure modes, no group displayed an irreparable fracture located one-third apical. The most prevalent failure mode across all groups consisted of unrepairable fractures at one-third from the root, occurring in 60% of the NP-2 group, 50% of the NP-BL and NP-MD groups, and 10% of the NP-0 group. Subsequently, the potential restorability of fractures at one-third coronal position showed the highest occurrence, with a frequency of 50% in the NP-0 group, 30% in the NP-BL group, and 20% in the NP-MD group. This particular failure mode was not observed within the NP-2 group. Concerning restorable fracture failures at the neckline, these did not manifest in the NP-MD group, but were present in the NP-2, NP-0, and NP-BL groups at rates of 40, 30, and 20%, respectively. A minor number of specimens exhibited recoverable fractures in the coronal one-third of the root, accounting for 30% in the NP-MD group and 10% in NP-0. However, this failure mode was absent in the NP-BL and NP-2 groups ( Fig. 8 ).

## Discussion

In a 2018 study, Ortiz-Ruiz et al 23 demonstrated differences in the organic and inorganic content between human enamel and dentin when compared to bovine dentition. While the overall dentin structures appear quite similar, human teeth contain a higher concentration of the mineral hydroxyapatite. Nakamichi et al conducted pioneering comparative studies between bovine and human dentition, and their findings have proven to be reliable in dental research. 24 Histochemical and anatomical studies have revealed striking similarities in mammalian teeth. Furthermore, adhesion to enamel and dentin exhibited no statistically significant differences. In general, these structural similarities enable us to interpret research results using the animal substrate. 25 26 27 28

In a prosthetic treatment of an endodontically treated tooth, the complexity of the coronal destruction can influence the prognosis. 29 The presence, even if partial, of a remaining coronal tooth structure, provides the ferrule effect, which has been extensively discussed in the literature for its clinical benefits. 18 29 30 31 With this in mind, and to gain a better understanding of the ferrule effect, this study focused on the effect of the remaining coronal tissue associated with a nonpost approach. Through finite element analysis (FEA), it was observed that there was no difference between the groups with and without a post in the different geometric preparations of the remaining walls. This finding is consistent with the study by Fokkinga et al, 31 in which the prosthetic restorations were clinically followed for up to 17 years, showing no difference in the survival probability of prosthetic restorations with or without an intraradicular post. The key factor for clinical longevity was related to the presence of remaining coronal structures. A similar finding was reported in the study by Santos Pantaleón et al 30 regarding immediate fracture resistance, stating that core-supported prosthetic restorations without a post have a good prognosis when associated with the presence of coronal remaining structure and not dependent on the post. Consistent with the literature findings, the FEA in this study demonstrated that significant differences occurred between the groups with complete ferrule and those without ferrule, and these differences were not associated with the use of posts, as shown in studies by Juloski et al. 29

A study 29 using FEA of teeth restored with posts and ceramic crowns showed that teeth with a ferrule exhibited smaller areas of stress concentration in the adhesive area compared to teeth without a ferrule. In the present study, the groups without a ferrule showed higher MPS at the cement–crown and cement–core interfaces. The in silico analysis also revealed tensile stress concentration in the middle third of the root in all models. This finding may be associated with the predominant failure mode observed in the groups.

The primary failure mode observed in groups with both partial and complete ferrules predominantly involved nonrepairable fractures occurring in the root region. Interestingly, these fractures manifested under loads significantly higher than those typically encountered during normal masticatory forces. This suggests that under lower loads, the primary failure modes for these groups predominantly comprised repairable and potentially repairable fractures. In contrast, within the group lacking a ferrule, the most prevalent failure modes were repairable fractures occurring at the cementoenamel junction of the restoration and potentially repairable fractures in the cervical third. 32 33 34 35 Notably, a substantial portion of the samples, amounting to 60%, experienced fractures under a relatively low load of 200 N, which is notably lower compared to the other experimental groups.

In 2016, Shamseddine and Chaaban conducted a study evaluating different geometries of remaining coronal walls in combination with intraradicular retainers. 36 They found no increase in fracture resistance with different geometries, and in some cases, it even impeded the flow of the cementing material. This finding contradicts the findings of the present investigation. Despite the scarcity of literature and information on the failure mode of upper central incisors with and without a ferrule, it was observed through fatigue testing and FEA that the presence of mesiodistal remaining walls behaved similarly to having a complete ferrule. There was no statistically significant difference between the complete ferrule group and the mesiodistal ferrule group in the stepwise stress test, and these values were consistent with the MPS findings obtained through stress maps. However, the NP-BL group had fatigue results similar to the NP-0 group, also corroborating the FEA findings. This is due to the significantly lower elastic modulus of dentin (18.6 GPa) compared to that of composite resin (21.62 GPa), resulting in the restoration having a biomechanically unnatural behavior making the root easily deformed or fractured. Furthermore, the dental enamel on the buccal surface is thinner than in other areas of the tooth, making it more susceptible to damage, such as repeated exposure to chewing forces. This means that this area is subject to repeated loads and shear forces that can cause wear over time.

The study by Elavarasu et al 37 demonstrated that a favorable prognosis would be to achieve a complete ferrule with a height of at least 2 to 3 mm. However, a literature review by Juloski et al 29 showed that in a clinical situation where it is not possible to obtain a complete ferrule, an incomplete ferrule is a better clinical option than the absence of it. Studies have indicated a high prevalence of caries in proximal contact areas, highlighting the difficulty in achieving a configuration of a complete ferrule. 30 31 37 In the present study, the group with a partial ferrule mesiodistal exhibited a biomechanical behavior similar to that of the complete ferrule group ( Table 1 ). Therefore, the present results confirm that having a partial buccal and/or palatal ferrule is preferable over the complete absence of a ferrule effect, despite the statistical results not differing significantly from the NP-0 and NP-BL groups.

When comparing the fatigue test with FEA, the region with the highest stress in all groups was the middle third of the root, followed by the cement–core and cement–crown interfaces, and the core itself. These results align with the failure modes observed after the fatigue test, where most failures occurred in the root region rather than debonding or core fracture. Another point to consider is that the presence of a post increases stress in the core. In 2018, a systematic review by Nauman et al reported that in clinical situations where there are no remaining coronal structures, no consensus or definitive finding supports or rejects the use of posts. 7 However, an *in vitro* study by Iemsaengchairat and Aksornmuang 38 demonstrated that in the cases where ETT have thin remaining root walls and a complete absence of a ferrule, cast metal posts and cores provided the highest fracture resistance, followed by multiple fiber posts and composite resin cores.

Certainly, it is crucial to acknowledge the potential limitations inherent in both in silico and *in vitro* studies, which often involve specific configurations and rely on idealized conditions simulated through mathematical analyses. 38 Consequently, there remains a need for clinical studies aimed at assessing the longevity and treatment success of ETT with varying degrees of remaining walls, with or without posts. Clinical research is indispensable in providing valuable insights into the real-world performance of these restorative approaches. It takes into account a multitude of factors, including professional experience, 39 patient-specific considerations, oral hygiene practices, occlusal forces, 40 and long-term treatment outcome. 41 42 By amalgamating the findings obtained from the current results with those emerging from further investigations, a holistic understanding of the biomechanical behavior and the clinical implications surrounding different remaining wall configurations can be attained. 39 43 This, in turn, holds the potential to refine treatment strategies and ultimately enhance patient outcomes.

## Conclusion

FEA revealed similar mechanical responses in models with and without posts, as well as among groups featuring total and partial ferrule tips in maxillary central incisors. Nevertheless, the highest survival rate was associated with the presence of a complete 2-mm ferrule. In the cases where achieving a complete ferrule is unfeasible, opting for an incomplete ferrule is recommended, with the ferrule's positioning being a crucial factor. Importantly, statistical analysis showed no significant differences between the groups with and without posts, regardless of the presence of a ferrule, particularly in the context of upper anterior teeth.
